# Dehydrogenation vs Apparent Hydrogenation: Unraveling the Mechanisms of He and O_2_ Plasma Etching on Colloidal Nanocrystal Films

**DOI:** 10.1021/acsami.5c14331

**Published:** 2025-11-10

**Authors:** Santosh Shaw, Tiago Silva, Jonathan M. Bobbitt, Fabian Naab, Cleber L. Rodrigues, Emily A. Smith, Ludovico Cademartiri

**Affiliations:** † Department of Materials Science & Engineering, Iowa State University of Science and Technology, Ames, Iowa 50011, United States; ‡ Instituto de Física, 28133 Universidade de São Paulo, São Paulo 05508-090, Brazil; § Department of Chemistry, Iowa State University of Science and Technology, Ames, Iowa 50011, United States; ∥ Michigan Ion Beam Laboratory, 1259 University of Michigan, Ann Arbor, Michigan 48109, United States; ⊥ Ames Laboratory, United States Department of Energy, Ames, Iowa 50011, United States; # Department of Chemistry, Life Sciences and Environmental Sustainability, University of Parma, 43012 Parma, Italy

**Keywords:** plasmas, colloidal nanocrystals, ligand stripping, thin films, ion beam analysis, Raman spectroscopy, etching, nanocrystal processing

## Abstract

Removing organic ligands from colloidal nanoparticles is critical for fabricating solid-state devices, yet accurately quantifying this removal remains a significant analytical challenge. Here, we establish a robust and accessible method for this quantification by calibrating Raman spectroscopy against precise ion beam analysis (IBA) for nanoparticle assemblies (CNAs) processed by helium (He) and oxygen (O_2_) plasmas. We demonstrate that the calibration curves are remarkably independent of plasma power and pressure, depending critically only on the choice of feed gas. He plasma induces rapid dehydrogenation and cross-linking, evidenced by a much faster decrease in the C–H Raman signal relative to the actual carbon loss. Conversely, O_2_ plasma leads to a surprising “apparent hydrogenation”, where the carbon backbone is removed significantly faster than the C–H signal diminishes. This counterintuitive effect is explained by a serial mechanism of oxidative fragmentation; β-scission cleaves the alkyl chains, and subsequent stabilization steps enrich the remaining film with hydrogen-rich methyl-terminated fragments, while carbon is efficiently removed as volatile CO. This work provides calibrated functions that enable the rapid determination of absolute carbon content in processed CNAs using simple Raman spectroscopy with uncertainties of ∼8% for O_2_ and ∼12% for He plasma, offering a vital tool for both process diagnostics and fundamental studies of plasma–matter interactions in colloidal nanocrystal films.

## Graphical abstract

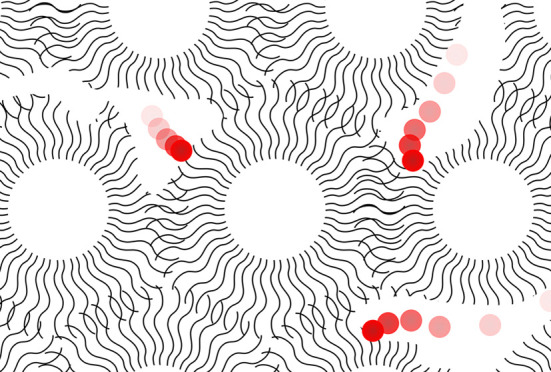

## Introduction

The application of colloidal nanoparticles in most solid-state devices will require their conversion to an inorganic solid-state material with a preserved nanostructure.^1–7^ This transformation requires the removal of the organic fraction from colloidal nanoparticle assemblies (CNAs), which can easily represent ∼40% of their volume.

This task is hard to accomplish and may be even harder to characterize. (i) Removing completely the ligands from CNAs is difficult due to mass transport limitations, the high reactivity of nanoscale inorganic surfaces, and the necessity to maintain the mechanical integrity of the assembly. (ii) Characterizing the removal of the ligands is challenging because adventitious carbon is ubiquitous^8^ and hard to account for, and instruments have either limited sensitivity for carbon (e.g., ^13^C NMR and EDX) or limited penetration depths (e.g., TOF-SIMS, XPS, and EELS).

Some of these challenges can be overcome by using sputtering in combination with a surface characterization technique (e.g., XPS or TOF-SIMS), but this approach is time-consuming and costly for the characterization of films that are thicker than 20–50 nm and can sometimes induce chemical changes in the material.^9^

There are a couple of different methods to remove ligands from colloidal nanocrystal assemblies: UV or UV–ozone treatments (preserve nanoparticle morphology but tend to leave behind residual carbon and can unintentionally oxidize the nanocrystal surface^10^), ligand stripping (can compromise structural integrity and leaves undesorbed organics^11^), and calcination (now established to be remarkably ineffective at the removal of organics in nanostructured materials^12^).

Plasma processing is an underexplored approach with significant advantages.^13^ (i) It can fully remove the organic fraction of the CNAs,^14^ even when using non-oxidizing gases, like He.^15^ (ii) The removal of the ligands is homogeneous throughout the thickness of the material,^14^ at least for films as thick as 400 nm. (iii) The processing occurs at relatively low temperatures (40–60 °C), therefore avoiding most diffusion and grain growth.^16,17^ (iv) The etching by O_2_ and He plasmas is highly selective to organics (extensive structural characterization in prior literature has shown no change on the structure of the nanoparticle cores,^14,18–20^ with the exception of potential surface oxidation^16,21^ ), which allows for the bottom-up control of solid/solid interfaces in CNAs by selective etching of ligands containing inorganic elements (e.g., trioctylphosphine oxide converts to phosphate groups by exposure to O_2_ plasmas).^17^ (v) Plasma processing is also scalable to large areas, it is a relatively green processing strategy due to the absence of solvents, and it can be used to produce crack-free all-inorganic materials from CNAs.^18^

While plasmas can be intimidatingly complex,^22^ their reproducibility is excellent (they have been in use industrially for decades); it is essential for researchers interested in using or studying the interaction of plasmas with CNAs to easily characterize the chemical changes that plasmas impart on CNAs.

Ion beam analysis (IBA) is a family of techniques that are ideally suited for this problem.^23^ They allow for highly quantitative, reproducible, accurate, and sensitive determination of elemental compositions, from hydrogen to uranium. Specifically, the sensitivity for carbon is ∼0.1 atomic % [or better with elastic recoil detection analysis (ERDA) or elastic backscattering spectrometry (EBS)] with an accuracy of ∼1%. The techniques can characterize in one shot all elements several micrometers of depth in the material, and by modeling the results, one can extract compositional profiles for each element measured with nanometer-scale resolution (typically 5–20 nm). Although the techniques are 50 years old at least, they still need specialists to run accelerators, set an experiment properly, and do the analysis of the spectra. Furthermore, they do not provide the same kind of chemical information that XPS or other techniques can provide.

Raman scattering can give chemical and structural information about both the organic and inorganic phases of the material. It can measure films that are nanometers to micrometers thick with a spatial resolution of a few hundred nanometers at best;^24,25^ most Raman spectroscopy experiments have a spatial resolution of a few micrometers.^26^ Unfortunately, amorphous phases of carbon generally have a small Raman cross section,^27,28^ which can easily lead to false negatives. However, with proper calibration, Raman spectroscopy can give absolute quantitative compositional information.^29–31^

Calibrating Raman data (e.g., the integral of Raman peaks) with IBA data (e.g., the carbon concentration in thin-film units, TFU, i.e., 10^15^ atoms/cm^2^) on the ligand removal from CNAs by plasma processing would combine many of the assets of IBA and Raman spectroscopy. The correlation between IBA TFUs and Raman peak areas should depend only on the etching mechanism and relies on a few assumptions: (i) the organic fraction of the ligands is mostly alkyl chains (which is true for the vast majority of ligands used in nanoparticle synthesis); (ii) the removal of the ligands is homogeneous throughout the thickness of the CNA (which was demonstrated before for films thinner than 450 nm^14^); and (iii) the nature of the inorganic phase does not influence the chemical mechanism of etching (e.g., through catalysis or mass transport limitations).

We here compare the Raman and IBA characterizations of the organic fraction of ZrO_2_-based CNAs as a function of the removal of the ligands (trioctylphosphine oxide) by both O_2_ and He plasmas. We find that Raman characterization of the ligand removal by He and O_2_ plasmas can indeed be calibrated by IBA data and that the calibration curve is largely independent of the plasma pressure between 100 and 2000 mTorr and power between 7 and 30 W despite the large differences these parameters cause on the etch rates.

The feed gas is the only plasma parameter that was found to significantly change the calibration curve. Specifically, He plasma causes significant dehydrogenation of the organic backbone (especially in initial stages of etching), leading to a fast decrease in the intensity of CH modes in Raman spectroscopy. O_2_ plasmas instead lead to an increase in the H/C ratio presumably due to carbon removal as CO and CO_2_ as well as fragmentation of the alkyl chains.

In summary, the calibration curve that we report enables the measurement of the absolute carbon concentrations in CNAs by simple Raman characterization with an uncertainty of ∼8% for the O_2_ plasma and ∼12% for the He plasma. This uncertainty is small enough to enable the systematic study of interactions of plasmas with colloidal nanocrystal assemblies and to allow for rapid diagnostic of ligand removal by plasma in CNAs.

## Experimental Design

ZrO_2_ nanoparticles [∼1.6 nm diameter, trioctylphosphine oxide (TOPO) ligands] were used for our study (Figure 1b). These were synthesized according to a published protocol.^32^ This material was chosen as a model system because it is easy to synthesize in a gram scale; it has superb colloidal stability^18^ (it can be thoroughly cleaned from excess ligands); and it is hard (which limits the influence of sputtering due to ion bombardment in the plasma). The nanoparticles were deposited from a highly concentrated hexane dispersion by spin coating on Si substrates, yielding disordered CNAs with a reproducible thickness of ∼370 nm. Disordered CNAs have been shown to avoid cracking induced by volume loss due to plasma processing^18^ as well as calcination.^33^

**Figure 1:**
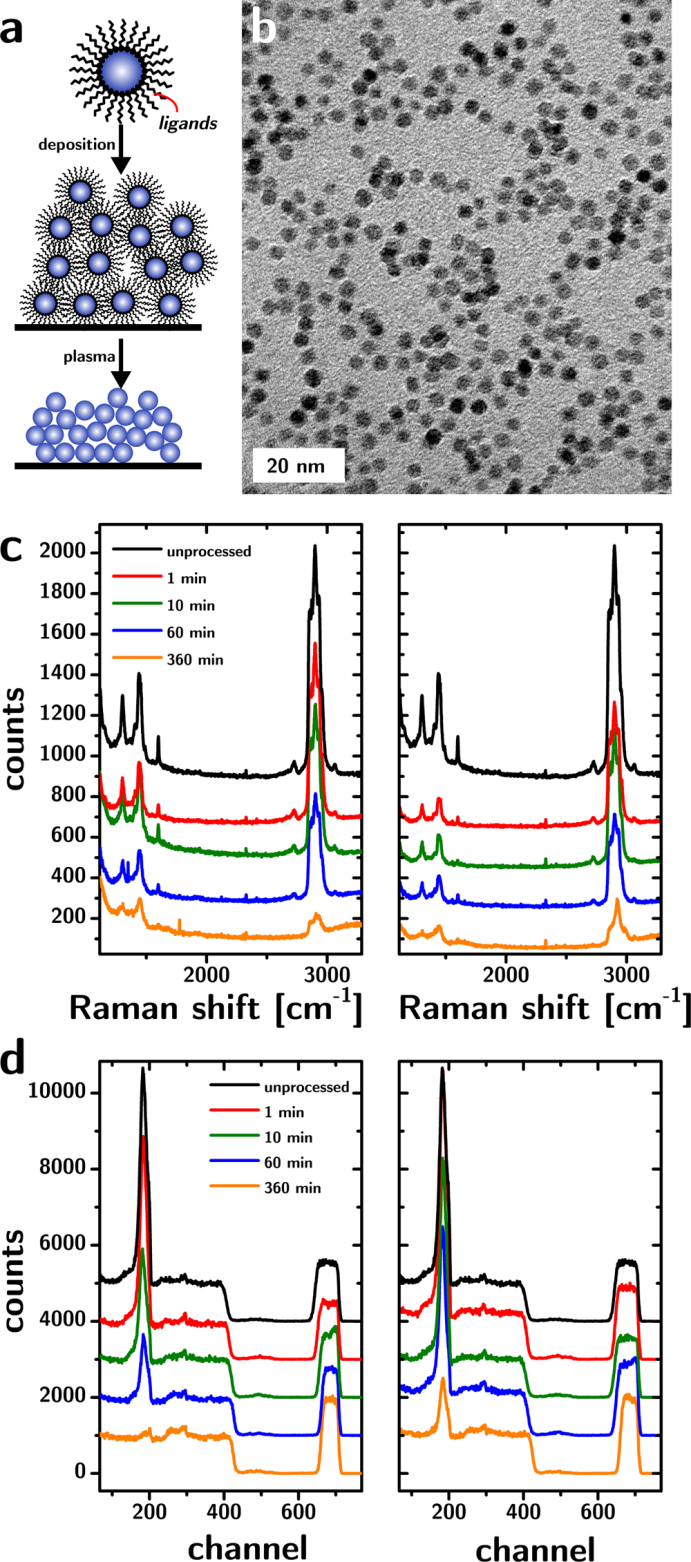
(a) Scheme describing the removal of ligands from colloidal nanoparticle assemblies, resulting in an all-inorganic nanostructured material. (b) Representative transmission electron microscopy (TEM) micrograph of the nanoparticles. (c) Raman scattering of the CNAs processed by the O_2_ (left) and He (right) plasmas as a function of the processing time. The drastic reduction in the intensity of the CH_2_ twist at 1370–1520 cm^–1^, the C=C bond at 1570–1630 cm^–1^, and the CH stretch peak at 2800–3000 cm^–1^ is particularly noticeable. (d) EBS characterization of the CNAs processed by the O_2_ (left) and He (right) plasmas as a function of the processing time, showing the significant drop in the carbon concentration (peak at channel 200). For clarity, panels c and d only show data from the samples processed by 30 W and 500 mTorr plasmas.

A XploRA Plus confocal Raman microscope with a 50 μm slit and a 100 μm pinhole (Horiba Scientific, Edison, NJ) was used to collect Raman spectra of the ZrO_2_ films. A 1200 grooves/mm grating set at a 2200 center wavenumber gave a spectral range from 900 to 3300 cm^–1^. The Raman spectra were collected for 120 s using 25 mW of a 532 nm excitation source. A 50× long working distance objective (Olympus, Waltham, MA) with a 0.5 numerical aperture was used to focus the laser onto the surface of the ZrO_2_ films. Peak areas were determined by integrating the CH_2_ (1370–1520 cm^–1^) and CH (2800–3000 cm^–1^) regions in the Raman spectra. The integrated areas were normalized by using integrated peak areas from an unprocessed film from the same batch.

To establish our correlation between IBA and Raman characterizations of ligand etching, we used inductively coupled plasma provided by a plasma cleaner (Harrick Plasma Cleaner PDC-001 with a PlasmaFlo gas flow mixer). This plasma cleaner is ubiquitous in university laboratories, is relatively inexpensive, and achieves the low power conditions that are necessary to prevent sputtering with the majority of feed gases (sputtering still happens if Ar is used as a feed gas). We explored a parameter space including three values of pressure (100, 500, and 2000 mTorr), two values of power (7 and 30 W, the “low” and “high” power settings of the instrument), two feed gases (O_2_ and He), and four processing times [1 min, 10 min, 1 h (60 min), and 6 h (360 min)].

## Results and Discussion

### Raman Characterization of CNAs

The Raman spectrum of the unprocessed CNA (Figure 1c) is consistent with peaks associated with TOPO as well as one additional peak (a reference spectrum for TOPO is provided in Figure S1 ^34^). Interestingly, a peak at 1600 cm^–1^ can be observed in the Raman spectrum, corresponding to C=C, which is not present in the bulk Raman spectrum of TOPO (spectrum not shown). It is known that ZrO_2_ supports are catalysts for dehydrogenation reactions,^35^ which might explain the formation of C=C in TOPO adsorbed to the CNA. Another (less likely but that cannot be dismissed) possibility is that the double bonds originate from adventitious carbon or impurities in the TOPO source. The Raman scattering from the CNAs shows the expected drop in the intensity of the CH_2_ twist (∼1450 cm^–1^) and CH stretch (∼2900 cm^–1^) modes with increasing processing times for both He and O_2_ feed gases, which is consistent with previous reports (cf. Figure 1c).^20,36^ The 1600 cm^–1^ Raman peak intensity also decreases for both feed gases and is not observable after 6 h of processing (Figure 1c).

### Correlation with IBA

The EBS spectra from the same films confirm the removal of carbon (peak at channel 200 in Figure 1d). The analysis of the EBS spectrum requires that the carbon peak be fitted with the rest of the spectrum to determine the concentration of each element in the system.

The peaks at 2800–3000 cm^–1^ (CH stretch) and 1370–1520 cm^–1^ (CH_2_ twist) in the Raman spectrum can be integrated to yield a quantitative parameter for use in the calibration. A bar graph of the 2800–3000 cm^–1^ peak area as a function of time is shown in Figure 2 for the 7 W and 2000 mTorr data set. At 60 min, the O_2_ plasma treatment has a statistically lower CH peak area compared to the He plasma treatment. The data for 7 W and 100 and 500 mTorr only shows a statistical difference between He and O_2_ plasmas at 1 min processing time for 500 mTorr. Similar trends are observed for the CH_2_ twist peak.

**Figure 2:**
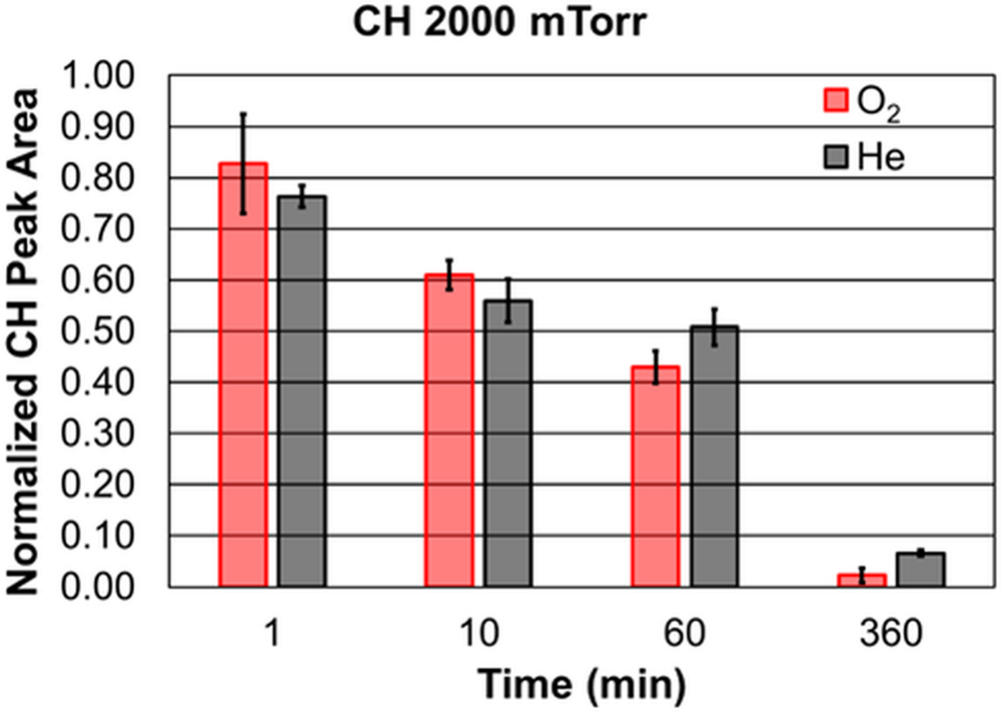
Bar graph of the normalized CH stretch peak area as a function of the processing time at 2000 mTorr for the O_2_ (red) and He (black) feed gases. There is a no statistical difference in the relative CH peak area until after 60 min of processing. The error bars represent the standard deviation or replicate measurements taken from three different spots for a given film.

The plot in Figure 3a allows us to compare Raman and IBA analyses and establish a correlation that could be used as a calibration. On the abscissa is plotted the area of the CH Raman peak, normalized against the corresponding peak area for the unprocessed CNA. On the ordinates are the carbon TFU values, also normalized against the value obtained from the unprocessed peak. Each data set is a time series of four CNAs that are processed in a certain set of plasma conditions (He feed gas = black scatters; O_2_ feed gas = red scatters; squares = unprocessed; circles = 7 W and 100 mTorr; up triangles = 7 W and 500 mTorr; down triangles = 7 W and 2000 mTorr; rhombi = 30 W and 100 mTorr; left triangles = 30 W and 500 mTorr; and right triangles = 2000 mTorr), for a total of 49 samples. Deviations within the samples were found to be very low in a previous study,^20^ and so each data set corresponds to a single measurement.

**Figure 3:**
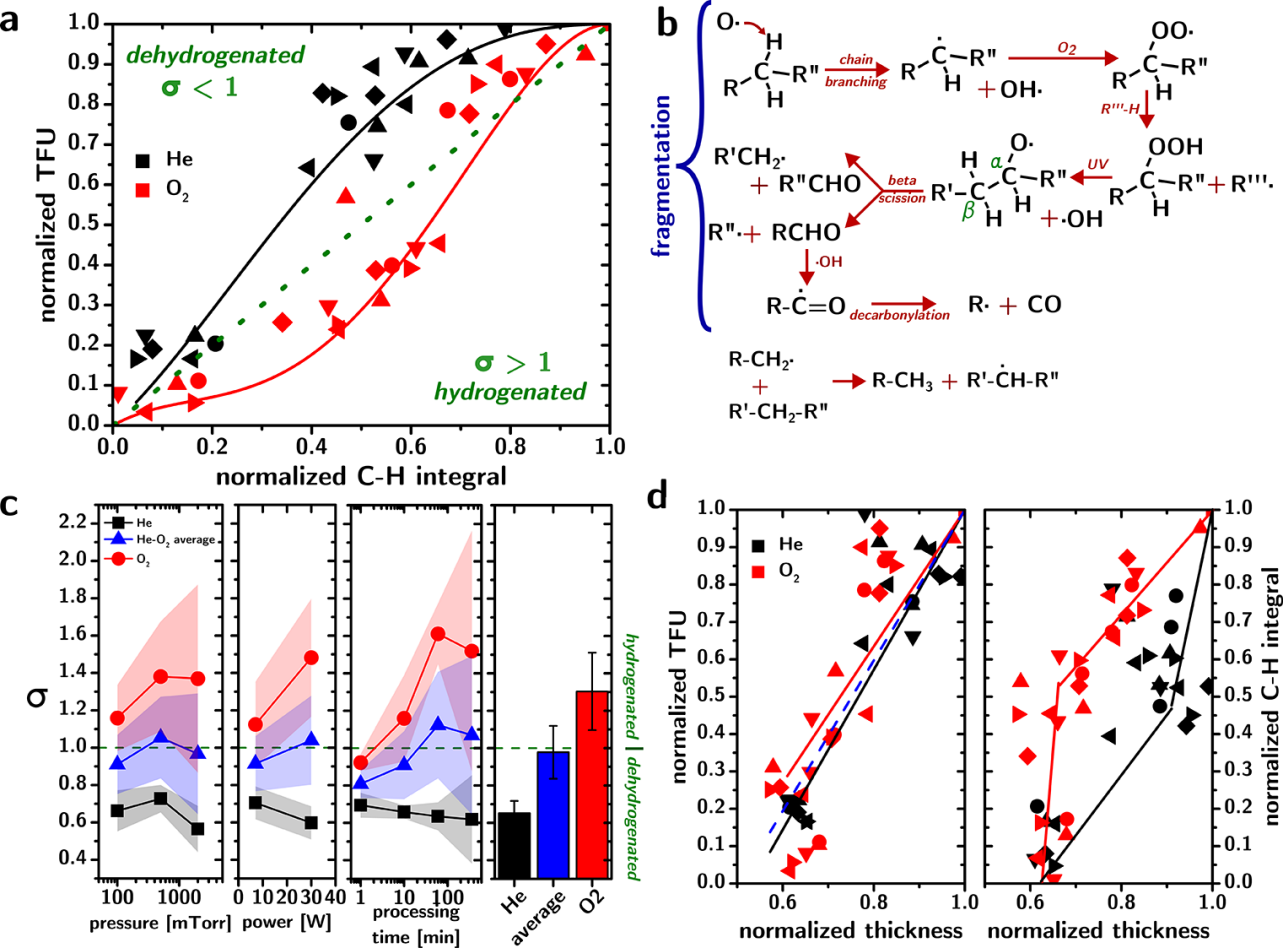
Comparison of Raman and IBA characterizations of plasma etching of CNAs. (a) Plot of carbon TFUs against the CH peak area, both normalized against the unprocessed CNAs, for CNAs processed by O_2_ (red) or He (black) plasmas. (b) Step by step oxidative fragmentation of alkyl chains in O_2_ plasma followed by ethyl to methyl conversion of terminal radicals explains the apparent “hydrogenation” of the organic fraction in CNAs upon O_2_ plasma. (c) Plots of the average σ values as a function of (from left to right) plasma pressure, plasma power, plasma processing time, and feed gas. (d) Plots of the correlation between the CNA thickness and normalized carbon TFU (left) or normalized CH peak area (right). In the left panel, the lines indicate linear fits of the data (red line for the O_2_ data; black line for the He data; and blue dashed line for all data), while on the right panel, the lines are guides to the eye.

### Feed Gas Dependence

The plot comparing Raman and IBA data can be separated into two groups corresponding to the two different feed gases; variations in pressure, power, and exposure time do not seem to have as much of an effect on the position on the plot as the feed gas.

The position of a data set on this plot is not devoid of chemical information. Since the unprocessed material is the point (1,1) in this plot, all points within the plot represent the removal of the ligand. Data along the diagonal should correspond to films whose average composition of the organic fraction could be comparable to that of the unprocessed CNA. Data that are off the diagonal indicate instead that the composition must differ from that of the unprocessed CNAs. For example, if the ligands lose more C–H bonds during etching than carbons due to cross-linking, the CH peak area would decrease faster than the carbon TFU count, leading to data sets above the diagonal. On the contrary, if the number of methyl groups is increased, for example, by the fragmentation of a cross-linked organic fraction, then the CH peak area will decrease more slowly than the carbon TFU count, leading to points beneath the diagonal line.

The plot in Figure 3a shows that the major influence of the plasma parameters on etching is due to the feed gas. In the case of He, the curve can be described as a saturation curve that stays above the diagonal for the entire range of the CH peak area. In the case of O_2_ plasmas, the curve is better described as a sigmoidal curve that appears to follow the diagonal at high CH integrals only to dip below it at a low CH peak area. The data suggest that He plasmas have a clear dehydrogenating effect on the carbon framework,^37^ consistent with the findings that we reported in a previous work,^15^ which supported the rapid cross-linking of the ligands due to heavy VUV and UV irradiation. O_2_ plasmas instead tend to remove carbon from the CNAs faster than they remove hydrogen; most of the data lie below the diagonal. This is an apparently extremely surprising result; how could a possibly ultra-aggressive oxidizing environment lead to an apparent “hydrogenation” of organic matter?

### Mechanistic Interpretation

The plausible resolution of this apparent contradiction is actually very much part of the understanding of plasma etching in O_2_ (cf. Figure 3b). We know from previous work that the etching of the organics in CNAs by plasmas is driven by a radical-initiated mechanism;^20^ we will not consider ablative or bombardment-initiated processes. The process begins when highly reactive species from the oxygen plasma, such as atomic oxygen or hydroxyl radicals, attack the long alkyl chains of the ligands. This initial strike creates a reactive carbon radical on the backbone.^38^ This radical site immediately engages in rapid oxidative reactions. It forms a peroxyl radical,^39^ which is then quickly converted into a highly unstable alkoxy radical.^40^ This alkoxy radical is central to the process, as it readily undergoes β-scission, which cleaves the carbon backbone. Each β-scission produces a stable, oxygen-containing molecule, such as an aldehyde, and a new, smaller end-chain radical (ECR).^41^ The aldehyde products are not stable in O_2_ plasmas but are subsequently attacked by OH radicals to form acyl radicals,^42^ which then decarbonylate to CO and a terminal radical.^43^

The ECRs produced by β-scission and decarbonylation stabilize themselves by intermolecular chain transfer via hydrogen abstraction.^44^ This crucial step produces a stable, hydrogen-rich, methyl-terminated fragment that remains in the film and a new radical on the neighboring chain, which becomes the next victim in the fragmentation cascade.

The organic material that survives and remains in the film to be measured by Raman spectroscopy is enriched in the fragments that successfully stabilized themselves. This creates the illusion of “hydrogenation” because the H-richer species are the survivors of this competitive process.

The overall reaction shown in Figure 3b can be summed up as (eq 1)­

1 $${R^{\prime }\mathrm{CH}}_{2}{R^{\prime \prime }+R^{\prime \prime \prime }H+O}_{2}=R^{\prime \prime \prime *}+R^{\prime \prime *}+R^{\prime *}+{\mathrm{OH}}^{*}+\mathrm{CO}$$

in the presence of O* and UV, which are intrinsic to the O_2_ plasma environment. Two of the three radicals produced at the end (R′* and R″*) are necessarily ECRs, which can easily stabilize into terminal methyl by hydrogen abstraction. None of the oxygen from the plasma is permanently added to the organic fraction, but rather carbon is lost by volatilization as CO. The overall effect is that stable organic matter gets progressively converted from ethyls to methyls, while carbon is removed, which is exactly what our data is compatible with.

The curves that we used to fit the He and O_2_ data sets are the following polynomials (eqs 2 and 3):

2 $$\left(\mathrm{for He plasmas}\right),\mathrm{TFU}=1.138\mathrm{CH}+2.21{\mathrm{CH}}^{2}-3.834{\mathrm{CH}}^{3}+1.486{\mathrm{CH}}^{4}$$

3 $$\left({\mathrm{for O}}_{2}\mathrm{plasmas}\right),\mathrm{TFU}=0.699\mathrm{CH}-3.189{\mathrm{CH}}^{2}+8.282{\mathrm{CH}}^{3}-4.791{\mathrm{CH}}^{4}$$

where TFU indicates normalized TFU concentrations and CH indicates normalized peak areas of the peaks associated with CH modes. The quality of the fits is adequate (0.89 for both the O_2_ and He sets) and allows one to extrapolate a carbon concentration value (in TFUs) from a Raman characterization of the CH peak if the unprocessed film is used as an internal standard. By analyzing the distribution of residuals from these fits, we estimate the error associated with values extrapolated from these calibration curves as ±0.08 for O_2_ and ±0.12 for He.

To better understand the information contained in the data, we define a parameter, called σ, which is defined for each sample *i* as σ_*i*_ = CH_*i*_/TFU_*i*_. For σ = 1, the organic composition of the sample is similar to that of the unprocessed film (at least in terms of saturation of the hydrocarbons). For σ > 1, the sample is hydrogenated and vice versa compared to the standard. Figure 3c shows the average values of σ for samples processed at different pressures, powers, and times and in different feed gases. The shaded areas behind the scatters identify the 95% confidence intervals on the average value. The histogram on the right shows how different the average σ is for O_2_ (σ = 1.30 ± 0.21) and He (σ = 0.65 ± 0.07) samples, consistent with the qualitative assessment made on Figure 3a. Increasing the plasma pressure or power only appears to strengthen the effect associated with the feed gas; i.e., the σ increase in σ of the O_2_ plasmas increases σ, while the He plasma decreases it. The same applies to longer processing times (the data from a long processing time have a large error due to the very low amounts of carbon left in the CNAs).

Future work employing isotopically labeled ligands could provide further confirmation of the hydrogenation/dehydrogenation pathways. However, the present correlation between IBA and Raman data already provides direct quantitative evidence for the different fates of carbon and hydrogen in O_2_ vs He plasmas.

### Thickness as a Proxy

As previously mentioned, thickness can also be considered an easily measurable proxy of the carbon concentration and, therefore, a candidate for calibration with IBA data. The calibration curve that results is shown in Figure 3d. Compared to the Raman calibration in Figure 3a, the thickness calibration has a much greater noise, which makes it less useful as such (without considering the significant fact that the Raman signal is not linear with thickness). Furthermore, as discussed previously, the change of thickness as a result of ligand removal would depend on a multitude of factors that are sample-specific.

The real value of the correlations between the thickness of the CNAs and the IBA (Figure 3d, left panel) and Raman characterization (Figure 3d, right panel) lies in the information they give regarding the morphological changes occurring in the film as a result of the specific etching process. The change in thickness of the film appears to be linearly related to the carbon TFUs in the film. Importantly, both the O_2_ and He data sets can be reasonably well fit with a straight line (*R* ^2^ = 0.7 for both sets). The global fit of all data (blue dashed line in Figure 3d) yields a slope that is not significantly different from that of the other fits; the O_2_ and He data sets do not appear to be significantly different in terms of the dependence of thickness to TFU. These data suggest that each TFU of carbon that is removed by the plasma leads to a specific loss of thickness of the CNA, regardless of what type of plasma is involved.

The picture is completely different and much more informative when looking at how thickness correlates with the CH peak area measured by Raman scattering (Figure 3d, right panel). Not only are the trends not linear, but the O_2_ and He plasmas lead to nearly specular behavior. In the O_2_ plasma, the initial decrease in the CH peak area is linearly dependent on thickness until a threshold value of relative thickness (∼0.7), below which the C–H peak area keeps decreasing without changing the thickness of the material. Instead, for He plasma, the initial remarkable drop of the CH integral is not accompanied by a very significant drop in thickness; for example, by the time the CH peak area is reduced by half, the O_2_ plasma samples have lost ∼40% of their thickness, while the He samples have only lost ∼10%. Below a normalized CH value of 0.4, the thickness decreases much more rapidly.

This nearly specular behavior of the two different feed gases suggests profound differences in the effect of etching on the structure of the CNA. The linear dependence of thickness on carbon TFUs quantified the loss in thickness associated with the loss of a certain amount of carbon. Therefore, the different behaviors of the CH peak area must be ascribed to the fate of hydrogen during the etching process.

The picture that emerges, consistently with the analysis of the σ factor, is that the He plasma leads to cross-linking^15^ of necks located between particles that are harder to reach. This results in an open structure with low carbon TFU and even smaller CH peak area but a relatively undiminished thickness. The change in behavior at a lower CH value might be due to the reduction in the rate of dehydrogenation (due to depletion of hydrogens in the plasma phase as well as in the organic phase, leading to a reduction in the rate of processes, such as H^•^ + RH → H_2_ + R^•^), causing the effect of He metastable species to become dominant, causing fragmentation of the carbon backbone and the formation of volatiles, by processes such as He* + R → R_1_^•^ + R_2_^•^ + He or R_1_^•^ + R_3_^•^ → R_1_R_3_ (↑).^37^

For O_2_ plasmas, the rapid decrease in thickness for a moderate loss in the CH peak area is consistent with a rapid fragmentation of the organic backbone,^45^ leading to the faster loss of ethylene groups than methylene groups and/or the rapid release of carbon as CO or CO_2_, therefore increasing the average H/C ratio and therefore the σ factor. This process leads to a drastic reduction of the thickness of the film until the fragmentation of the backbone reduces in rate, and a further loss of carbon by etching is accompanied by significant dehydrogenation through processes such as −C–C– + O → –^•^C–C– + ^•^OH or RH + 2O → R^•^ + H^•^ + O_2_.^45^

## Conclusion

We have here compared the quantification of carbon concentrations by ion beam analysis and Raman scattering. The study served two purposes: First, we provide the means to use Raman spectroscopy (a readily available, rapid characterization technique) for the quantitative measurement of the absolute concentration of carbon in colloidal nanocrystal assemblies that have been processed by O_2_ or He plasmas. By using IBA as an internal calibration standard, we find that the functions describing the correlation between carbon TFUs and Raman scattering from the CH modes are nonlinear but robust (i.e., only the feed gas significantly changes function).

Therefore, upon calibration with one IBA measurement, the curves provided should allow the characterization and estimation of the absolute carbon content of CNAs after plasma treatment with either the O_2_ or He plasmas. The error in the use of these calibration curves is estimated as 8% for O_2_ plasma and 12% for He plasma, at least within the range of plasma processing parameters that we explored.

The other goal of this study was to refine our understanding of the chemical and structural effect of O_2_ and He plasmas on CNAs. The correlation between carbon concentrations, thicknesses, and intensities of the Raman scattering from CH modes indicates that the two feed gases lead to drastically different chemical and structural transformations of the ligand shells during their etching. Early stages of etching in O_2_ plasmas lead to rapid carbon loss, while He plasmas lead to a rapid loss of hydrogen.

These data allow the exploration of the interaction of O_2_ and He plasmas with colloidal nanocrystal assemblies for both routine diagnostic purpouses (e.g., how much carbon is still left in the CNA?) and fundamental studies (e.g., what are the rate-limiting processes in the removal of ligands from CNAs?).

## Supplementary Material



## Notes

Reference Raman spectrum for trioctylphosphine oxide (Figure S1) (PDF)
