# Supplementary material for: Dehydrogenation vs Apparent Hydrogenation: Unraveling the Mechanisms of He and O2 Plasma Etching on Colloidal Nanocrystal Films
Source: ACS Appl Mater Interfaces. 2025 Nov 10;17(46):63707–14. doi: 10.1021/acsami.5c14331 (PMC12635958; doi:10.1021/acsami.5c14331)
Supplement: Supplementary file 1 [file am5c14331_si_001.pdf]

SUPPORTING INFORMATION

DEHYDROGENATION VS APPARENT HYDROGENATION: UNRAVELING THE MECHANISMS OF HE AND O_2 PLASMA ETCHING ON COLLOIDAL NANOCRYSTAL FILMS

SANTOSH SHAW[1], TIAGO SILVA[2], JONATHAN M. BOBBITT[3,5], FABIAN NAAB[4], CLEBER L. RODRIGUES[2], EMILY A. SMITH[3,5], LUDOVICO CADEMARTIRI[6]*

[1] Department of Materials Science & Engineering, Iowa State University of Science and Technology, Ames, IA, 50011, USA
[2] Instituto de Física, Universidade de São Paulo, São Paulo, 05508-090, Brazil
[3] Department of Chemistry, Iowa State University of Science and Technology Ames, IA, 50011, USA
[4] Michigan Ion Beam Laboratory, University of Michigan, Ann Arbor, MI, 48109, USA
[5] Ames Laboratory, U.S. Department of Energy, Ames, IA, 50011, USA
[6] Department of Chemistry, Life Sciences and Environmental Sustainability, University of Parma, Parma, 43012, Italy

[*] Author to whom correspondence should be addressed: ludovico.cademartiri@unipr.it

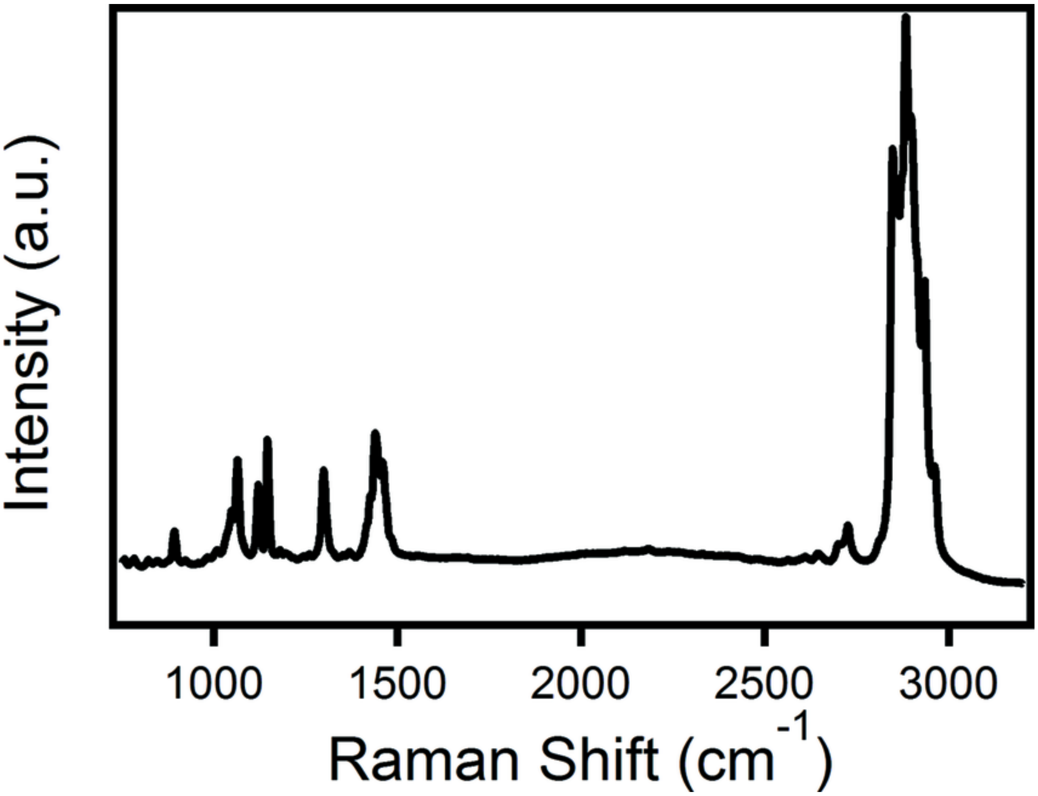

Figure S1. Reference Raman spectrum for TOPO. Reproduced with permission of the International Union of Crystallography [1]

References

1. Doan-Nguyen, V.; Carroll, P.; Murray, C., Structure determination and modeling of monoclinic trioctylphosphine oxide. *Acta Crystallographica Section C* **2015,** *71*, 239-241.
